# Miasmas, mental models and preventive public health: some philosophical reflections on science in the COVID-19 pandemic

**DOI:** 10.1098/rsfs.2021.0017

**Published:** 2021-10-12

**Authors:** Trisha Greenhalgh

**Affiliations:** Primary Care Health Sciences, University of Oxford, Oxford OX2 6GG, UK

**Keywords:** COVID-19, public health, philosophy of science, miasma, randomized controlled trial, evidence-based medicine

## Abstract

When the history of the COVID-19 pandemic is written, it is likely to show that the mental models held by scientists sometimes facilitated their thinking, thereby leading to lives saved, and at other times constrained their thinking, thereby leading to lives lost. This paper explores some competing mental models of how infectious diseases spread and shows how these models influenced the scientific process and the kinds of facts that were generated, legitimized and used to support policy. A central theme in the paper is the relative weight given by dominant scientific voices to probabilistic arguments based on experimental measurements versus mechanistic arguments based on theory. Two examples are explored: the cholera epidemic in nineteenth century London—in which the story of John Snow and the Broad Street pump is retold—and the unfolding of the COVID-19 pandemic in 2020 and early 2021—in which the evidence-based medicine movement and its hierarchy of evidence features prominently. In each case, it is shown that prevailing mental models—which were assumed by some to transcend theory but were actually heavily theory-laden—powerfully shaped both science and policy, with fatal consequences for some.

## 1. Mental models and the theory-versus-data question

As Coveney and colleagues have argued previously in this journal [1], the relative importance of empirical measurements (data) versus mental models (theory) has been a central preoccupation of philosophers of science since the days of Immanuel Kant, who famously observed that ‘Thoughts without content are empty, intuitions without concepts are blind’ [2]. This dictum is often interpreted to mean that science requires not just data but also the development and testing of mental models of causality (i.e. theories), though philosophers may argue that this is not strictly what Kant meant. Using big data as an example, Coveney *et al.* warn that however large and accurate the dataset, theory-free data-dredging will not provide meaningful or useful answers to scientific questions.

Empirical observation and measurement has long been viewed as the cornerstone of scientific inquiry and as a route to uncovering—or at least approaching—the truth. The Vienna Circle of logical empiricists (sometimes called logical positivists), for example, were concerned about the distortion of science by political and ideological forces (especially, at the time, Nazism) [3]. Members of the Circle adhered to the principle of *verificationism*—that only statements which are empirically verifiable are cognitively meaningful [4]. Through objective empirical study, they believed, science would transcend the distortions of thinking that came from metaphysics (literally, ‘beyond physics’—as they saw it, things constructed by the mind rather than things we can see and measure in the natural world).

While most of us would agree that ideology has no place in science, that is not the same as saying science can manage without mental models. Some philosophers of science have insisted that to measure something without seeking to understand and explain it is not science at all. As Sir Peter Medawar observed in his essay *Induction and intuition in scientific thought* [5], for example, scientists need to do more than ‘browse over the field of nature like cows at pasture’. This is because scientific reasoning is not merely the apprehension of facts but ‘an exploratory dialogue that can always be resolved into two voices: imaginative and critical’, hence ‘the initiative for scientific action comes not from the apprehension of facts but from an imaginative preconception of what might be true’ [5]. In Medawar's view, mental models and empirical data keep each other in check—he described them respectively as the ‘bride’ and ‘groom’ of science—and scientific progress in any discipline occurs by the back-and-forth dialogue between their two ‘voices’.

This view of science is compatible with the thinking of Thomas Kuhn (1922–1996), who introduced the notion of scientific paradigms—‘universally recognized scientific achievements that, for a time, provide model problems and solutions for a community of practitioners’ [6]. In other words, for any given scientific discipline, there is an agreed set of concepts and how they fit together, based on particular mental models of causality, which informs the framing and prioritization of research questions and how scientists should go about answering these questions. Science, Kuhn proposed, progresses within paradigms by the accumulation of data and refinement of theory, and—more radically—via the dialectical replacement of one paradigm by another when prevailing concepts, theories and methodologies become inadequate to address emerging questions, breakaway scientists form a new paradigm with different mental models and novel methodologies.

Other scholars of the scientific process, notably Bourdieu [7] and Knorr-Cetina [8], have theorized the paradigmatic differences between groups of scientists in more overtly political terms, noting that dominant mental models in science are linked to power (hence, influence) and resources. But the science–politics axis is a subject for another day [9]. In this paper, I want to focus on *philosophical* differences among groups of scientists in the relative emphasis they give to empirical data versus theory. I will contrast the assumptions and actions of scientists who assume that their experiments require little or no theory with those of scientists who develop and demand explicitly articulated theories to inform data collection and analysis. I will argue that all science is theory-laden and that trouble tends to emerge when empiricists claim to have transcended theory.

## 2. Cholera: miasma or waterborne?

One historical example of prevailing mental models and the influence that went with them is the extended length of time it took to replace the miasma theory of cholera (the assumption that it spread via the smell of sewage) with a waterborne theory in the mid-nineteenth century. Johnson [10] has described how public health experts of the day, notably the distinguished scientist and social reformer Sir Edwin Chadwick, were—at least for a time—convinced of the miasma theory. Policy decisions were explicitly built on this assumption. For a period in the 1840s and 1850s, cesspits in towns were banned and house and street refuse channelled directly into rivers. Physician John Snow imagined differently. He noted that cholera was transmitted among people who shared the same water supply, rather than people who shared the same air, and first published this information in 1849 [11]. But the statistical data gathered on deaths from cholera tracked only the factors that miasmists hypothesized to be important: elevation of the land, for example, because it was believed that miasma stayed low to the ground. However, in 1853 William Farr, who published the statistics in his *Weekly Returns* (lists of deaths) was interested in Snow's theory, so he added a new category: where victims got their water.

When a severe cholera outbreak occurred in Soho in 1854, Snow famously mapped the deaths to the water supply and showed that they could be traced to a single contaminated water pump in Broad Street; he was helped by local resident Henry Whitehead, who—using what has been termed ‘boots-on-the-ground epidemiology’—visited every house in the area and asked people where they had obtained their water in the days before the outbreak. Whitehead and Snow discovered that the physical distance from house to pump was not always useful, because some people deliberately walked further to their favourite pump, and some people had access to boiled water (via the brewery).

As is widely known, Whitehead and Snow got the handle of the Broad Street pump removed, quickly ending that particular cluster of cases. But as Johnson [10] describes, the same day the pump handle was removed, the national Board of Health ordered an investigation into the Soho outbreak—which was (unconsciously, I suspect) informed by their assumptions about miasma. They chose to look, for example, at such things as ventilation, smells (and whether people had complained about them), cleanliness of the houses, air temperature, weather conditions, whether the water looked and smelled clean and whether the containers used were clean. They did not ask which pump the victims had used, nor did they investigate whether the pumps could have been contaminated with water flow from elsewhere.

The authorities concluded that there was no evidence cholera was waterborne. As quoted in a relatively recent reprint [12]:

it has been suggested by Dr Snow, that the real cause […] lay in the general use of one particular well, situated at Broad Street in the middle of the district, and having (it was imagined) its waters contaminated with the rice-water evacuations of cholera patients. After careful enquiry, we see no reason to adopt this belief. We do not find it established that the water was contaminated in the manner alleged; nor is there before us any sufficient evidence to show, whether inhabitants of the district, drinking from that well, suffered in proportion more than other inhabitants of the district who drank from other sources [13].

This excerpt illustrates the powerful impact of an assumed theoretical model of causality on scientific thinking (and thence to policy decisions). The local public health authorities already ‘knew’ the mechanism of spread, so they found ‘no evidence’ to support an alternative theory—partly because they failed to look for it and partly because they overlaid empirical evidence with their existing mental model. It is not entirely clear why they believed that their own theory fitted the data obtained—but as Barnes & Bloor [14] observed, theory is underdetermined by data (in other words, the same data may be explained by multiple theories), so fit with one's preferred theory is not a sound reason for rejecting an alternative theory.

Using the rhetoric of scholarship (“after careful inquiry…”), but without actually rebutting Snow's arguments, the authorities of the day depicted Snow as unrigorous and mistaken. Academic peers were equally scathing in their reviews. While removal of the Broad Street pump handle is now seen as a landmark intervention in the history of public health, at the time the editor of the *Lancet* commented that ‘in riding his hobby [horse] very hard [Dr Snow] has fallen down through a gully-hole and has never since been able to get out again’ [15]. The two-sentence notice of Snow's death in the *Lancet* in 1858 did not even mention his contribution to cholera [16]. It was not until the devastating cholera outbreak of 1866, when 93% of the dead were customers of the contaminated East London Water company, that the miasma theory of cholera transmission was finally rejected by mainstream science and Snow's waterborne theory revisited, tested and accepted [10,16].

## 3. Evidence-based medicine: a method-focused, theory-light hierarchy of evidence

Those tempted to view the snail's pace march of science in nineteenth-century public health as a phenomenon of yesteryear should first consider how empiricist-dominated mental models continue to both shape and constrain science. One such model—evidence-based medicine (EBM)—has had particular influence in the COVID-19 pandemic. In the early 1990s, a group of epidemiologically trained doctors—the forerunners of the EBM movement—challenged the traditional way clinical decisions were made (essentially, mechanistic reasoning based on accumulated case knowledge) and introduced decision-making based on empirical evidence from randomized controlled trials (RCTs)—carefully controlled experiments in which participants are randomly allocated to ‘intervention’ and ‘control’ groups with systematic follow-up and measurement of predefined quantitative outcomes [17].

Initially seen as bold and heretical, EBM quickly became the new orthodoxy. Its declared mission was to strengthen medicine's empirical foundations while reducing its reliance on theoretical reasoning [18]. Central to its claim to legitimacy was the unique role of the RCT in reducing *bias*—that is, influences that could ‘deviate the results or conclusions … systematically away from the truth’ [19]. ‘Truth’ was seen as external and ascertainable through experiment and observation—and most especially, by doing RCTs *correctly* by following agreed methodological procedures. There was little or no recognition that facts are theory-laden and truth perspectival—for example, as scientists’ analytic gaze, and the questions they choose to ask, are shaped by the mental models with which they approach and communicate about the world [20].

EBM has long promoted a *hierarchy of evidence* with RCTs and meta-analyses of RCTs at the top and the so-called lesser forms of evidence below it [21]. This is, in reality, a hierarchy of empirical study designs, based on the assumption that some designs (notably, RCTs) are inherently more likely to bring scientists closer to the truth. An international network of EBM-inspired scientists, the Cochrane Collaboration, has developed a weighty handbook of instructions, based largely on technical criteria and checklists, for correctly undertaking and synthesizing RCTs [22].

Not everyone who aligns broadly with the EBM movement agrees that method should always and necessarily be privileged over theory. Indeed, there is a rich seam of philosophical writing on the need for EBM's ‘gold standard’ RCTs to be informed by causal explanations [23–25], and a recognized if somewhat niche sub-discipline within EBM is the study of mechanisms of causality [26–28]. Notwithstanding this literature, the maxim that researchers should develop and test theories of causality alongside undertaking RCTs, or that policymakers should take account of *explanations* when selecting and interpreting empirical evidence, is a rule that is honoured more in the breach than in the observance. The World Health Organisation's published summary of its guideline development process, for example, emphasizes EBM's hierarchy of evidence, RCTs and the over-riding need to eliminate bias, but makes no reference to explanatory theory [29].

The hierarchy of evidence, and the meticulous methodological rules and procedures developed to extend it, saved many lives during the pandemic. They facilitated the generation, at impressive speed, of definitive evidence from RCTs and meta-analyses on the efficacy and safety of drugs and vaccines [30,31]. Given that new drugs and vaccines may have toxic side effects which could conceivably be worse than the disease itself, it was wholly appropriate to require objective empirical evidence from RCTs of the benefit–harm balance before such products were approved for widespread use.

But as the next section describes, the same rules and procedures—and the empiricist assumptions which underpinned them—were used to justify a more questionable approach to research on the effectiveness of preventive measures such as facemasks in controlling spread of the virus.

## 4. The contested efficacy of facemasks in preventing spread of SARS-CoV-2

Along with other non-pharmaceutical interventions, facemasks were included in a Cochrane systematic review, *Physical interventions to interrupt or reduce the spread of respiratory viruses*, originally published in 2011 [32]. When the pandemic was declared, that review was rapidly updated and placed on a preprint server [33]. Unlike the 2011 version, the update excluded all evidence on facemasks except 14 RCTs, each of which had been systematically assessed for ‘risk of bias’ before being included in a meta-analysis. Only one of the 14 included trials related to use of masks by the lay public to prevent community transmission (the others related mainly to masks worn by healthcare workers in a professional setting), and that study was not in COVID-19. Since the meta-analyses did not reach statistical significance, the authors’ conclusion was that there was no firm evidence that facemasks work. They called for larger, better-designed RCTs.

The style and tone of the Cochrane review was what Bourdieu [7], following Gilbert and Mulkay, called the ‘empiricist repertoire’ [which] is characteristic of formal experimental research […]: the style must be impersonal and minimise reference to social actors and their beliefs so as to produce all the appearances of objectivity; reference to the dependence of the observations on theoretical speculations disappear; everything is done to mark the scientist's distance from his model; the account given in the ‘methods’ section is expressed in the form of general formulae…’. Yet in a highly accessed accompanying blog, the review's authors added some somewhat speculative theorizing about potential harms which revealed mechanistic—and empirically unsubstantiated—assumptions about a droplet mode of spread and risk compensation behaviour: ‘thinking you're protected means that you will put yourself at higher risk’ and that ‘[y]ou may also end up touching your face more often’ [34].

A group of Danish researchers, also hospital doctors trained in the EBM tradition and Cochrane Collaboration members, sought to fill the evidence gap flagged by Jefferson and Heneghan in their Cochrane review. They designed a masks-on versus masks-off RCT, which—after some months’ delay—was published in a leading medical journal [35]. The trial found no statistically significant difference in COVID-19 incidence in people who wore facemasks compared to those who did not. But it had numerous flaws. It was, for example, underpowered (i.e. too small by an order of magnitude to test its main hypothesis), conducted at a time when the incidence of COVID-19 was very low, had an intervention period of only one month (woefully inadequate given that the antibody test used took more than two weeks to turn positive after an infection), and addressed whether facemasks protected the wearer rather than the more important question of source control—whether they prevent transmission to others [36]. Despite these flaws, the Danish study was hailed in some circles as definitive evidence that facemasks ‘do not work’.

Both the Jefferson systematic review and the Heneghan–Jefferson commentary seem to reflect a mental model of extreme caution when introducing new treatments. But compared to a new drug or vaccine, the risk of serious harm from facemasks is extremely low, and the potential for benefit at population level could be high. Hence, it could be argued that the usual reasons for advocating caution in clinical trial research do not hold. Indeed, because of the very different balance of probabilities, there are strong arguments for reversing the usual assumption that avoiding harm is more important than striving for benefit. We should, perhaps, adopt the precautionary principle and recommend this intervention ‘just in case’ [37]. In philosophy of science terminology, this is sometimes known as the problem of inductive risk [38].

## 5. Philosophical challenges to the randomized controlled trial

The assumptions which placed the RCT in a bias-free class of its own have rightly been challenged [39]. Particularly when evaluating complex social interventions such as lockdowns, school closures, physical distancing or the wearing of masks to protect other people, a study design that requires the random allocation of people to intervention and control groups and their follow-up to measure particular predefined outcomes may be impractical, unethical, unacceptable, underpowered, overly narrow, insufficiently nuanced, impossible to undertake ‘blind’, or unable to generate definitive results either at all or within the required timeframe [40].

But there is a more fundamental—i.e. philosophical rather than methodological or practical—objection to the emphasis on RCTs *to the exclusion of other kinds of evidence*, and that is the assumption, based on what might be called naive empiricism, that data can be identified, collected, analysed and summarized without the need for theory. Academics in many other scientific disciplines emphatically reject the assumption that controlled experiments should always and necessarily over-ride *mechanistic evidence*, defined as evidence produced by multiple different methods which help illuminate and explain phenomena at a theoretical level [40,41].

Mechanistic evidence is, arguably, not *inferior* but *complementary* to evidence from RCTs (Medawar's ‘bride’ and ‘groom’). In order to refine an intervention to maximize its potential impact, we need to understand, at a theoretical level, the chain of causality linking intervention to outcome. While well-conducted RCTs may have high internal validity (i.e. they can produce strong evidence for the population from which the sample was drawn), their external validity may be low (i.e. their findings may not apply to other populations or settings). Mechanistic evidence allows scientists to elucidate the different steps in the causal pathways that help us anticipate why an intervention which worked in one setting is also likely work in a different setting—and also to reason why an intervention that did *not* work in one setting could still have an important contribution to a programme of interventions somewhere else.

For all these reasons, to build a robust knowledge base about interventions, and depending on the precise circumstances, it is sometimes necessary to draw on a wide range of evidence, both mechanistic and experimental, and use review methods that do not merely *summate results*, as in the Cochrane Collaboration's empirically driven systematic reviews [22], but also *explain mechanisms* and *enrich our understanding*, as in more theory-driven narrative reviews [42,43]. Ogilvie and colleagues use the telling metaphor of the brick wall for the Cochrane systematic review: every contributory RCT is a brick; ideally, all bricks should be the same in terms of the research question addressed, outcome measures used, and so on [43]. Brick by brick, the selected-for-similarity primary studies make their respective contributions to an overall grand mean through meta-analysis. By contrast, Ogilvie *et al.* depict the synthesis of mechanistic studies (the more heterogeneous the better) to produce a sense-making narrative review as the building of a dry stone wall—an artisan craft in which each stone is carefully selected to make a unique contribution to fill a particular shaped gap.

I undertook, with colleagues, a literature review on the benefits and possible harms of facemasks [37]. Our review methodology aligned with the dry stone wall metaphor, covered a vast array of mechanistic evidence including laboratory studies of aerosolization, natural experiments across different countries and regions, case studies of super-spreader events, qualitative studies of people's attitudes in different cultural contexts, and computer modelling studies. This review had limitations—for example, it fell short of a systematic examination of evidence for and against a range of different hypotheses. But within those limitations, we identified sparse, moderate and strong evidence from the different streams listed above—but no disconfirming evidence—that facemasks are effective in preventing community spread of SARS-CoV-2, and no evidence whatsoever that they cause serious harm. In addition, by drawing on mechanistic evidence, we were able to explain apparent discrepancies in the data, especially the ‘negative’ findings of the Danmask RCT.

## 6. A miasma theory of SARS-CoV-2 spread?

During 2020, in an ironic reversal of the paradigmatic battle between airborne (miasma) and waterborne explanations for the spread of cholera in the nineteenth century, key advisory groups including the WHO, US Centers for Disease Control and Prevention, European Centre for Disease Control and Public Health England all assumed that the dominant mode of transmission of SARS-CoV-2 was respiratory droplets. Just as in the 1850s, policymakers assumed the mode of transmission rather than seeking empirical demonstration of it. They dismissed claims from people who argued that the virus was—or could be—significantly spread through the air. And they ignored what philosophers of science might call ‘black swan’ evidence (i.e. real-world observations that cannot be explained by prevailing mental models) such as super-spreader events. The ill-fated performance of Bach's St John Passion in Amsterdam's Concertgebouw auditorium in March 2020, for example, soon after which 102 of 130 choir members developed symptoms of COVID-19 and four people died, simply cannot be explained by an exclusively droplet mode of transmission [44].

Droplets emitted in coughs and sneezes are relatively heavy and fall to the ground or onto surfaces quickly. A droplet mode of spread means that transmission of the virus will occur only when in close proximity to others—1 m according to the WHO; 1.5 or 2 m according to other bodies—and also via our hands (which are easily contaminated by droplets, for example when we touch our eyes, nose or mouth) and fomites (objects we may touch with contaminated hands, such as our mobile phones). ‘Contact and droplet mitigation strategies’ (preventive efforts built on droplet theory) include the physical distancing of shoppers in queues plus frequent and assiduous washing of hands and wiping-down of surfaces. Masks, according to the droplet hypothesis, need only be worn when physical distancing is impossible, and are viewed as a relatively minor component of the prevention package.

Despite the WHO's firm insistence on the droplet theory of transmission, this mental model was not universally accepted. Indeed, in China where the disease had emerged, the prevailing theory was based on recent historical experience with the SARS and MERS viruses, both of which had been shown to be airborne [45]. An airborne model of spread means that transmission occurs *both* at close range *and* at longer distances (via ‘shared air’), and that the chemistry of air composition, the physics of air flow and the architecture of the built environment are all influential in the model of spread. Using this mental model, masks, worn by everyone at all times when inside a building or vehicle, were a key factor that could keep the air virus-free—which is why, even before 2020, many people in Asian countries wore masks outside the home. Japan's highly successful ‘3Cs’ prevention policy—avoid crowded places, closed spaces and close contact—was based on an assumed airborne route of transmission [46].

But in the West, and throughout most of 2020, policy thinking was dominated by the empiricist conclusion that there was ‘no evidence’ for the efficacy of masks and by speculation, based on a droplet model of spread, that both risk compensation and touching the mask could cause harm [34]. Tellingly, these statements were not corrected even when empirical evidence emerged demonstrating no risk compensation [47] and no increase in face-touching [48] in people who wore facemasks.

Because different mental models informed very different policies in different countries, a vast natural experiment resulted. The findings were striking. Countries which had recommended facemasks for the public in the first 30 days from the first documented case (usually because key authorities accepted a miasma—airborne—theory of spread) had, on average, orders of magnitude fewer deaths from COVID-19 than countries which delayed such recommendations beyond the first 100 days [49]. But just as in the 1850s, publication of this mechanistic evidence led, in many settings, not to the immediate adoption of a new mental model but to a doubling-down on the old model.

## 7. Policy will ‘follow the science' of policymakers' mental models

The WHO's position on prevention of COVID-19 is based largely on advice from its Infection Prevention and Control Research and Development Expert Group for COVID-19. Most of its members are clinicians with a background in hospital-based infectious diseases and training in EBM; they are experts in topics such as wound management—for which droplet spread is predominant and handwashing is an effective intervention [50]. Throughout 2020, this group appear to have engaged only to a limited extent with the considerable volume of mechanistic evidence available at the time that the SARS-CoV-2 virus is airborne and is spread significantly if not predominantly by tiny aerosols (particles between 5 and 100 µm in diameter which account mainly for short-range transmission but which can travel several metres—beyond the limits of physical distancing measures) [51,52]. Speaking and singing, which produce few droplets, generate large numbers of aerosols. These reviews also offered evidence that fomites are unlikely to be a major route of transmission because almost everyone who has attempted to culture the virus *from surfaces* has been unsuccessful and the virus can remain viable *in air* for several hours and that under certain environmental conditions (notably, cold, poorly ventilated and extremes of humidity) it can travel many metres and persist for hours. They also argued that airborne transmission is strongly suggested by well-documented super-spreader events (such as singing performances) and nosocomial outbreaks (within healthcare facilities). The conclusions of these early reviews have been affirmed and strengthened by more recent summaries of the evidence [53,54].

Back in June 2020, over 200 aerosol scientists from around the world published an open letter addressed to international policymaking bodies summarizing studies undertaken by its signatories which had demonstrated ‘beyond any reasonable doubt’—so long, one might add, as one accepts the validity of mechanistic evidence—that the SARS-CoV-2 virus is released in tiny microdroplets small enough to be carried long distances in the air when people talk, cough and even just exhale [55]. Yet a few weeks later, WHO committee members published an article expressing the view that the virus ‘is not spread by the airborne route to any significant extent’ [56], a conclusion that was quickly challenged by post-publication peer review [57]. Mental models appear to have led to two errors by the paper's authors: dismissal of a new and potentially plausible theory because it failed to resonate with their methods-focused privileging of RCTs and rested heavily on mechanistic evidence which they did not value; and the logical error of conflating what they took to be *lack of evidence in favour* of aerosol spread with *evidence refuting* aerosol spread.

## 8. Distinguishing mental models from ideology and intellectual rigidity

While the Vienna Circle of logical positivists did not reject theoretical reasoning altogether, they placed little emphasis on it and identified some kinds of theoretical reasoning as potentially sinister and anti-science. They sought, for example, to purge science of German romanticism, a mental model which speculated that there was something pure and good about ‘German blood’, as they saw it serving the cause of political and ideological groups (specifically, justifying genocide) [3]. But the empiricist quest to produce a science free of ‘metaphysics’ in order to avoid the misuse of science by ideologically motivated movements was philosophically misplaced, since it—arguably—did not distinguish sufficiently between theories which are scientific (i.e. testable mental models of something that could be the case) with those that are non-scientific (things that cannot be tested or have already been shown empirically to be flawed). Medawar [5] reminded us that it is the *possibility of truth* that distinguishes the scientific imagination from the fanciful. The notion of the purity of German blood, for example, is a theory of sorts but not a scientific one, whereas waterborne transmission of cholera is a scientifically testable theory.

During the COVID-19 pandemic, libertarian groups drew heavily on what they took to be objective empirical data (especially the Danmask trial) and rejected mechanistic explanations based on indirect—and, they felt, low-quality—evidence. To a greater or lesser extent, people who aligned with the libertarian movement took the view that recommendations to stay home, maintain 2 m distance, wear facemasks and even get vaccinated were unwelcome intrusions of the state. They believed that segmentation should be practised instead of lockdown (that is, the old and vulnerable should stay at home in order that the young and less vulnerable could enjoy their freedoms and remain economically productive), that facemasks were harmful and an unacceptable infringement of personal freedom, and that this essentially mild disease should be allowed to wash over the population to achieve what was termed ‘herd immunity’ [58]. This combination of views, combined with distortions of Christian doctrines, proved particularly toxic in parts of the USA [59].

One explanation for why ideology and mental models became entangled in this example is that the Danmask RCT represented the closest to gold-standard evidence in the mental models that prevailed among the EBM community—namely, it used a study design from the top of the hierarchy of evidence and was ‘unbiased’. The libertarians were simply ‘following the science’ when they seized on the Danmask trial. But this does not explain why the trial's evident flaws were not acknowledged by many senior members of the EBM community. The Danmask trial, for example, was published without a CONSORT statement—the internationally agreed methodological checklist required by many journals as a condition for accepting a RCT for publication [60]. Unusually, the study's authors did not involve a clinical trials unit when designing their study, which may explain what some would describe as elementary flaws in its design. These near-universal checks and balances were, for some reason, not viewed as needed for this particular study.

A second explanation, then, is that the flawed RCT evidence on facemasks was either wilfully or unconsciously misinterpreted. Because one possible interpretation of its findings is that facemasks have no effect, ideological mental models came to align with scientific hypotheses in the minds of some libertarians (both lay and academic), who quickly deemed those hypotheses to be supported by ‘robust’ (i.e. RCT) evidence. While Medawar's writings imply that a scientific hypothesis can be readily distinguished from the fanciful, the facemask example suggests that this is not universally the case.

A reviewer of an earlier draft of this paper suggested that opposition by some individuals to the evidence on the efficacy of facemasks might be explained more in terms of sheer stubbornness than by recourse to mental models. This comment raises the question of whether the tendency to mistake ideological hypotheses for scientific ones may stem from intellectual vices. As Quassim Cassam has observed, academic effort can be understood in terms of intellectual virtues—defined as aspects of mind that promote effective and responsible intellectual inquiry, such as carefulness, flexibility, open-mindedness, conscientiousness and creativity—and intellectual vices—defined as aspects of mind that inhibit effective and responsible intellectual inquiry, such as excessive conformity, carelessness, rigidity, prejudice, closed-mindedness, dogmatism, complacency and arrogance [61]. The urgency of the pandemic, and the profound threats to our lives and lifestyles from both the virus itself and proposed containment measures, have brought out both the best and the worst in scientists. Both ‘sides’ in the facemask debate have been accused, by supporters and critics, respectively, of exhibiting both virtues and vices.

## 9. Conclusion

In this paper, I have argued that both the long delays in replacing flawed, miasma-driven approaches to cholera prevention in the nineteenth century and long delays in replacing an exclusively contact-and-droplet model of SARS-CoV-2 prevention with one that includes airborne transmission in the twenty-first both had a philosophical explanation in terms of which mental models of reality prevailed and the extent to which scientists and policymakers favoured data over theory. In the more recent example, ideological movements in the West drew—eclectically—on statements made by scientists, especially the confident rejection by some members of the EBM movement of the hypothesis that facemasks reduce transmission.

The WHO changed its stance to recommend the more extensive use of facemasks by the lay public in December 2020 [62]. By early 2021, it had begun to talk about the importance of ventilation—but at the time of writing, its advice is still focusing largely on contact and droplet measures such as surface cleansing and handwashing, and explicitly privileging the bricks of RCT evidence over the odd-shaped dry stones of mechanistic evidence.

While I disagree with the scientists who reject the airborne theory of SARS-CoV-2 transmission and the evidence for the efficacy of facemasks, they should not be dismissed as ideologically motivated cranks. On the contrary, I believe their views are—for the most part—sincerely held and based on adherence to a particular set of principles and quality standards which make sense within a narrow but by no means discredited scientific paradigm. That acknowledged, scientists of all creeds and tribes should beware, in these fast-moving and troubled times, of the intellectual vices that tempt us to elide ideology with scientific hypothesis.

## Data accessibility

All data referred to in the manuscript are in the public domain as published papers.
